# International Conference on Superlattices, Nanostructures and Nanodevices (ICSNN 2010)

**DOI:** 10.1186/1556-276X-6-82

**Published:** 2011-01-12

**Authors:** Shushen Li, Jian Liu, PingHeng Tan

**Affiliations:** 1 State Key Laboratory for Superlattices and Microstructures, Institute of Semiconductors, Chinese Academy of Sciences, Beijing 100083, China

## 

This special issue of *Nanoscale Research Letters *contains scientific contributions presented at the International Conference on Superlattices, Nanostructures and Nanodevices (ICSNN 2010), which was held in Beijing, China from July 18-23, 2010.

ICSNN 2010, attended by more than 260 participants from 24 countries and regions, is the sixteenth meeting in this biannual conference series. ICSNN 2010 was devoted to a continuation of the efforts by the research community to understand new phenomena in the physics and technology of superlattices, nanostructures and nanoscale devices, to highlight contributions from young scientists in this field, and to explore unconventional ideas in these areas that get insufficient attention at many conferences.

There are thirty papers in this special issue, covering a wide range of research fields including nanostructure fabrication techniques, nanoelectronics, graphene, quantum dots, quantum wells, spinelectronics, and magnetic/semiconductor hybrid nano-systems.

We would like to express our appreciation to all members of the International Advisory and Program Committees for their invaluable suggestions and selection of the invited speakers and scientific contributions. Our special thanks go to Professor Jean-Pierre Leburton for his advice to the local organizing committee.

We also thank the authors for their excellent contributions, as well as the referees whose feedback and comments ensured the high quality of this special issue.

We are looking forward to the next conference in 2012!

Conference Chairman

Shushen Li, Institute of Semiconductors, Chinese Academy of Sciences, China

Local Organizing Chairman

Jian Liu, Institute of Semiconductors, Chinese Academy of Sciences, China

Guest Editors

Shushen Li, Institute of Semiconductors, Chinese Academy of Sciences, China

Jian Liu, Institute of Semiconductors, Chinese Academy of Sciences, China

PingHeng Tan, Institute of Semiconductors, Chinese Academy of Sciences, China

